# Clinical profile of cerebral venous sinus thrombosis and the role of imaging in its diagnosis in patients with presumed idiopathic intracranial hypertension

**DOI:** 10.4103/0301-4738.60092

**Published:** 2010

**Authors:** Prateek Agarwal, Mahesh Kumar, Vipul Arora

**Affiliations:** Department of Neuro Ophthalmology, Aravind Eye Hospital and Post Graduate Institute of Ophthalmology

**Keywords:** Cerebral venous sinus thrombosis, idiopathic intracranial hypertension, magnetic resonance imaging, magnetic resonance venography

## Abstract

Retrospective descriptive study reporting the rate of occurrence of cerebral venous sinus thrombosis (CVST), highlighting the role of magnetic resonance imaging (MRI) and magnetic resonance venography (MRV) in patients with presumed idiopathic intracranial hypertension (IIH). Study was conducted in the department of neuro-ophthalmology at a tertiary eye care center in South India. Data from 331 patients diagnosed with IIH from June 2005 to September 2007 was included. Inclusion criteria were: Elevated opening cerebrospinal fluid (CSF) pressure of more than 200 mm of water on lumbar puncture, normal CSF biochemistry and microbiology, and normal neuroimaging as depicted by computed tomography(CT) scan. Exclusion criteria were: Space-occupying lesions, hydrocephalus, meningitis, intracranial pressure within normal range, abnormal CSF biochemistry and microbiology. The remaining patients were evaluated with MRI and MRV. CVST was present in 11.4% of patients who were presumed to have IIH (35/308). MRI alone identified 24 cases (68%) of CVST, while MRI used in combination with MRV revealed an additional 11 cases (32%). Risk factors associated with CVST were identified in nine out of 35 patients (26%). CVST may be misdiagnosed as IIH if prompt neuroimaging by MRI and MRV is not undertaken. Risk factors of CVST may not be apparent in all the cases and these patients are liable to be missed if CT scan alone is used for neuroimaging, hence MRI, combined with MRV should be undertaken to rule out CVST.

The clinical spectrum of CVST closely mimics that of idiopathic intracranial hypertension (IIH). IIH is mainly a diagnosis of exclusion based on Dandy's criteria:[1] Presence of elevated intracranial pressure (ICP) on lumbar puncture, normal cerebral spinal fluid (CSF) biochemistry and microbiology, no localizing sign with the exception of abducens nerve palsy and normal neuroimaging as depicted by computed tomography (CT) scan. Since Dandy's criteria were formulated prior to the MRI era[2] CT scan alone may be an insufficient tool for accurate diagnosis of IIH. MRI, along with MRV wherever necessary should be the modality of neuroimaging for accurate diagnosis.[3,4]

Risk factors for cerebral venous sinus thrombosis (CVST) may not be apparent in all the cases, hence it is difficult to exclude CVST clinically in these patients. Magnetic resonance imaging (MRI) in combination with magnetic resonance venography (MRV) is recommended to correctly diagnose CVST in these patients.[2–4]

We report the rate of occurrence of CVST in patients with presumed IIH as well as the associated risk factor profile, which prompted subsequent MRV.

## Materials and Methods

The study was conducted in the department of neuro- ophthalmology at a tertiary eye care center in South India. Patients diagnosed with IIH between June 2005 and September 2007 were included in this study. Inclusion criteria were: Elevated opening CSF pressure of more than 200 mm of water on lumbar puncture, normal CSF biochemistry and microbiology, and normal neuroimaging as depicted by CT scan.

Patients were excluded if they had space-occupying lesions, hydrocephalus, meningitis, ICP within normal range, abnormal CSF biochemistry and microbiology or bilateral disc edema from other causes like ischemic optic neuropathy, diabetic papillopathy. The remaining patients were evaluated with MRI, and MRV. MRV was performed only when MRI was not supportive in diagnosis of CVST, or there was strong clinical suspicion of CVST due to associated risk factors, namely male sex, non-obesity, post-surgery, use of oral contraceptives, deep vein thrombosis, hypercoagulable states in the presence of normal MRI.

## Results

Data of 331 patients diagnosed with IIH was retrieved. Twenty-three patients were lost to follow-up and hence excluded. Thirty-five patients out of 308 (11.4%) were found to have CVST. The study population included 240 females and 68 males. Mean age was 31.4 years (standard deviation 10.6 years).

**Figure 1:**
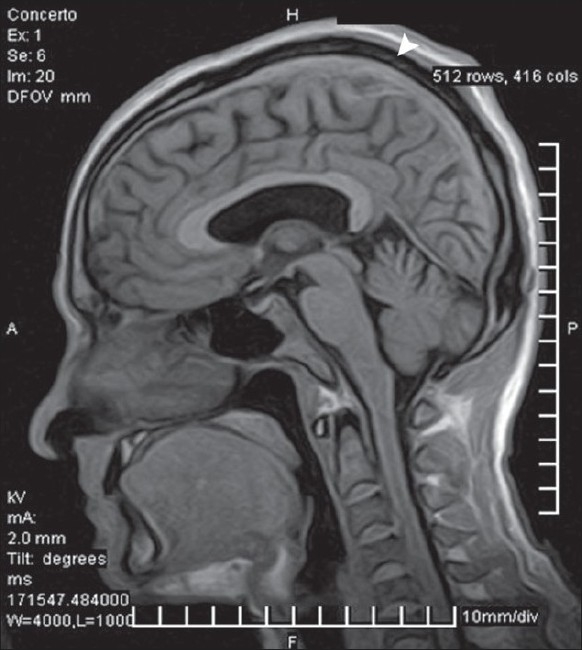
T1-weighted saggital magnetic resonance imaging showing hypointense lesion along the superior saggital sinus with abrupt discontinuity of the smooth contour, diagnostic of venous sinus thrombosis

**Figure 2:**
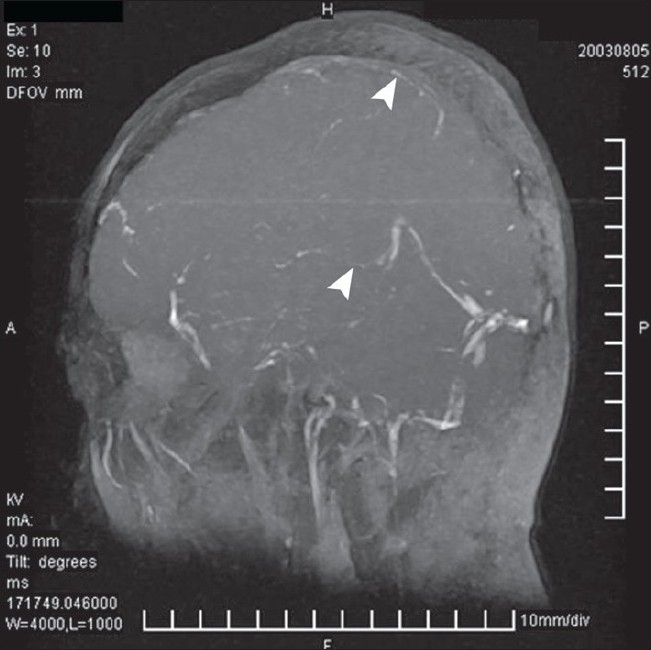
Magnetic resonance imaging showing flow signal void along the saggital sinus and transverse sinus, diagnostic of venous sinus thrombosis

**Figure 3:**
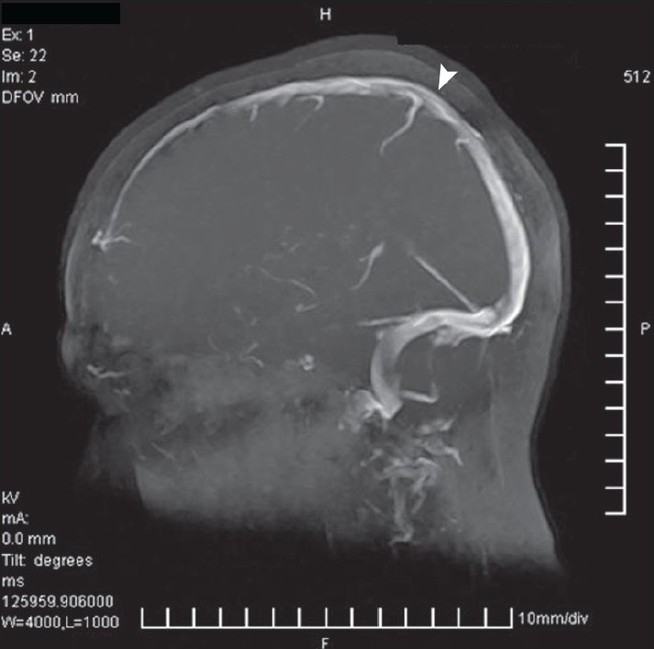
Normal magnetic resonance imaging showing complete filling of venous sinuses without any signal void

Among the CVST group there were nine females and 26 males (age range 17-58 years). Though MRI was diagnostic in 24 patients out of 35 (68%) showing hypointense lesion [Fig. 1] in the venous sinuses it was suggestive/suspicious in the remaining 11 cases. MRV showing signal void was diagnostic of CVST in all these 11 cases [Fig. 2]. Fig. 3 shows normal MRI for comparison. Thirty-one patients had thrombosis involving the superior sagittal sinus (88%). Four patients had isolated involvement of sigmoid and transverse sinus.

Underlying risk factors in the form of pregnancy (n = 2) post caesarean section (n = 2), deep vein thrombosis (n = 1), oral contraceptives (n = 2), hyperhomocystenemia (n = 1), leukemia (n = 1) were present only in nine cases (26%). Patients were referred to the neurosurgeon and were treated with anticoagulants, and acetazolamide. None of the patients with negative MRI/ MRV developed CVST in the subsequent follow-up of one year.

## Discussion

The clinical presentation of CVST closely overlaps that of IIH. IIH is a diagnosis of exclusion according to Dandy's criteria[1] i.e. symptoms and signs attributable to the raised ICP, abnormal CSF pressure on lumbar puncture, normal CSF composition, normal neuroimaging results, awake and alert patient and absence of localizing findings on neurological examination.

The dynamics of ICP are described by the relationship:[5,6]

Intracranial pressure = cerebrospinal fluid _FR_ × R_O_ + P_SS′_ where

Cerebrospinal fluid _FR_ = cerebrospinal fluid formation,

R_O_ = cerebrospinal fluid outflow resistance, and

P_SS_ = outflow pressure in the superior sagittal sinus.

It is apparent from this relationship that sagittal sinus hypertension will cause a concomitant increase in ICP.

The type of neuroimaging has to be modified in view of associated risk factors for CVST, namely male sex, non-obesity, use of oral contraceptives, deep vein thrombosis, hypercoagulable states, lupus anticoagulant, infections of ear, nose and throat, mastoiditis and history of surgery in the region of head and neck. It is crucial to differentiate CVST from IIH because the management protocols are entirely different. CVST is typically treated with anticoagulants and IIH with diuretics. CVST is life-threatening as it can cause stroke and death, unlike IIH, which stops with optic atrophy.

The combination of MRI and MRV is the diagnostic modality of choice for CVST.[7,8] MRV alone may be false-positive in cases of sinus aplasia or hypoplasia (seen as a flow gap). It can also mistake T2-weighted hypointense signal of deoxyhemoglobin and intracellular methemoglobin as flow void.[9]

The dural sinuses most frequently affected are superior sagittal sinuses, transverse and sigmoid sinuses.

Our results differ from the study of Lee *et al*.,[10] a smaller study that reviewed MRV results in 22 consecutive patients, all women who fit the typical demographic profile of IIH, and identified none of the patients with CVST. Purvin *et al*.[11] suggested that clinical manifestations of CVST may be differentiated from IIH by the abrupt onset and marked severity of symptoms.

However, the presentation of CVST can vary considerably based on the extent and location of thrombosis. The presentation of most of our patients with CVST was not the same as that described by Purvin *et al*. and closely mimics that of IIH.

Our study identified CVST in 11.4% of patients who were presumed to have IIH, and in the absence of correct diagnosis and lack of MRI and MRV these patients might continue to get treated as IIH, resulting in dismal visual prognosis, as well as significant risk of stroke and death. MRV in combination with MRI is recommended to identify this subgroup of patients who present with associated risk factors.

## References

[1] Dandy WE (1937). Intracranial pressure without brain tumour- diagnosis and treatment. Ann Surg.

[2] Sylaja PN, Ahsan Moosa NV, Radhakrishnan K, Sankara Sarma P, Pradeep Kumar S (2003). Differential diagnosis of patients with intracranial sinus venous thrombosis related isolated intracranial hypertension from those with idiopathic intracranial hypertension. J Neurol Sci.

[3] Vogl TJ, Bergman C, Villringer A, Ein Haupl K, Lissner J, Felix R (1994). Dural sinus thrombosis: Value of venous MR angiography for diagnosis and follow-up. AJR Am J Roentgenol.

[4] Biousse V, Ameri A, Bousser MG (1999). Isolated intracranial hypertension as the only sign of cerebral venous sinus thrombosis. Neurology.

[5] Borgesen SE, Gjerris F (1987). Relationships between intracranial pressure, ventricular size and resistance to CSF outflow. J Neurosurg.

[6] Hakim S, Venegas JG, Burton JD (1976). The physics of the cranial cavity, hydrocephalus and normal pressure hydrocephalus: Mechanical interpretation and mathematical model. Surg Neurol.

[7] Wang AM (1997). MRA of venous sinus thrombosis. Clin Neurosci.

[8] Sajjad Z (2006). MRI and MRV in cerebral venous thrombosis. J Pak Med Assoc.

[9] Connor SE, Jarosz JM (1997). Magnetic resonance imaging of cerebral venous thrombosis. Clin Neurosci.

[10] Lee AG, Brazis PW (2000). Magnetic resonance venography in idiopathic pseudotumor cerebri. J Neuroophthalmol.

[11] Purvin VA, Trobe JD, Kosmorsky G (1996). Neuro-ophthalmic features of cerebral venous obstruction. Arch Neurol.

